# ‘They Talk about the Weather, but No One Does Anything about It’: A Mixed-Methods Study of Everyday Climate Change Conversations

**DOI:** 10.3390/ijerph21030279

**Published:** 2024-02-28

**Authors:** Carl A. Latkin, Lauren Dayton, Abigail Winiker, Kennedy Countess, Zoé Mistrale Hendrickson

**Affiliations:** Department of Health, Behavior and Society, Bloomberg School of Public Health, Johns Hopkins University, Baltimore, MD 21205, USA; ldayton2@jhu.edu (L.D.); awinike1@jhmi.edu (A.W.); kcounte1@jhmi.edu (K.C.); zhendri1@jhu.edu (Z.M.H.)

**Keywords:** climate change, activism, collective action, barriers, communication

## Abstract

Understanding everyday conversations about climate change may provide insights into framing the issue to promote climate change action. As part of a longitudinal online study in the US launched in June 2021, 805 respondents were asked if they had discussed climate change with a friend or family member in the prior month; if not, why not, and if yes, they were asked to delineate the conversation topic. Concurrent mixed methods were used to analyze the data. The majority (62.6%) of respondents reported not having a conversation about climate change in the prior month. Among those who indicated that they had discussed climate change, five themes were identified from the conversation topics, with many having reported discussing the impact of climate change on weather patterns. Very few discussed actions to address climate change, and most of these discussions focused on individual-level behaviors rather than collective actions. Among participants who had not recently discussed climate change, the most prevalent theme was that it was not a priority or an issue they cared about. Results suggest that conversations may not lead to collective actions and that policymakers and environmental organizations should provide guidance on effectively channeling climate change concerns into action.

## 1. Introduction

According to the World Health Organization, “Climate change is the single biggest health threat facing humanity” [1]. The majority of the US adult public believes that climate change is occurring. A 2021 poll reported that 76% of respondents believed in the occurrence of global climate change, 70% were very worried or somewhat worried about global warming, and 55% believed that people in the US were currently being harmed by global warming [2]. However, only a small proportion of the public engages in political or collective actions to address climate change [3]. Many of those who are engaged in climate change actions focus their efforts on individual pro-environmental behaviors, such as reducing their personal fossil fuel use and recycling. Yet, individual-level actions are unlikely to significantly impact climate change without massive policy changes to curtail greenhouse gas emissions drastically [4,5]. Enacting such major policy changes is likely to require significant increases in the amount and intensity of collective action. Collective action can be fostered through social networks and social norms that promote the acceptability and importance of community engagement [6]. Conversations about climate change among peers may be a tool for fostering social norms of engaging in climate change activism and promoting collective action [7].

Recent studies on climate change communication have documented programs to teach students how to talk about climate change, parents’ interests in learning how to talk to their children about climate change, and framing climate change conversations with older adults [8,9]. Recommendations have also been formulated on how mental health clinicians can address and talk about climate change to reduce distress among their patients [10]. There is, however, limited research on peer-to-peer communication about climate change.

### 1.1. Peer and Public Climate Change Communication

Prior research on peer-level communication about climate change has frequently focused on how to convince network members that climate change is a threat and anthropogenic, and there is evidence that talking about climate change is linked to increased belief in the severity and anthropogenic cause [11]. However, there is little information about the frequency or content of the general public’s climate change conversations with family and friends, or whether these conversations focus on collective climate actions. There is also limited information about those who do not engage in interpersonal communication about climate change and the perceived barriers to engaging in these conversations. However, Howard [12] found that some environmentalists do not engage in climate change conversations due to fears of harming relationships. Understanding the content of climate change conversations and barriers to engaging in these conversations may be useful in developing messages and methods to facilitate peer communication about climate change that can lead to meaningful collective climate actions.

Climate change communication can sustain climate change concerns and potentially drive action. Brulle, Carmichael, and Jenkins [13] examined the drivers of public concern over global climate change across nine years and found that attitudes shifted over time. The authors found that scientific information on climate change had a limited impact on changing public opinion, and media coverage was found to increase concern about climate change, but only for short durations. The authors thus determined a need for “continuous public communication efforts to maintain support for climate change action in the face of opposing messaging campaigns”. In addition, Galway and colleagues [14] found that talking to family and friends about climate change mediated the relationship between connectedness to nature and individual-level climate action. As friends and family members are key sources of social influence, it is important that individuals engage in conversations about climate change. Geiger et al. [15] reported that civic discussions increase self-efficacy for future discussions of climate change, and van Swol [16] found that intimacy in group discussions increases climate change consensus. Yet, to have a meaningful impact on policies to mitigate climate change, these discussions should highlight the need to engage in collective action to mitigate climate change, such as supporting advocacy groups and lobbying political leaders to address climate change. However, it is difficult to know how to steer conversations to maximize their chances of facilitating climate change actions and advocacy without understanding the content of current climate change conversations.

Identifying demographic differences in engaging in recent climate change conversations may also help in the tailoring of messages to promote climate change action. Conversation topics may be influenced by a range of demographic and social factors. For example, analyses of political conversations suggest that political conversations are less likely to occur among Latinx individuals compared to white individuals and are more frequent in higher-education neighborhoods than in low-education neighborhoods [17,18].

The content of political leaders’ climate change discourse is also likely to influence the content of climate change conversations. This dynamic may occur directly through leaders’ public discourse or indirectly through their political tactics. For example, Donald Trump’s public hostility toward climate change science and addressing climate change had a substantial impact on his followers’ attitudes. Zawadzki et al. [19] found that in an online sample surveyed before and during the Trump administration, supporters’ climate beliefs grew weaker, whereas opponents’ climate beliefs grew stronger after his election. Additionally, a study on the impact of Pope Francis’ climate change encyclical, Laudato Sí, and his visit to the United States, during which he discussed climate change, found that among respondents who were already concerned about climate change, greater exposure to the Pope’s climate change message was linked to increases in attitude–behavior consistency in willingness to join a campaign to convince elected officials to take action to reduce global warming [20].

### 1.2. Framing Climate Change Discourse

Individuals and organizations have different approaches to framing and addressing climate change. Discourse analysis has been used to examine how the media, governments, and corporations have framed climate change issues [21]. Chater [22] highlighted how the Conservative government in Canada de-emphasized the impact of climate change on humans. Kapranov [23] analyzed the framing of climate change by fossil fuel corporations in Europe and the US. He found that Statoil and other European companies included an anthropogenic cause frame, which was not observed among US fossil fuel companies. In a study of Flemish newspapers, Moernaut and Temmerman [24] developed a typology of climate change frames, including six anthropocentric subframes and four biocentric subframes, and linked different frames to the articles’ news value. A study of Irish newspapers identified seventeen affirmative normative statements (i.e., statements about what should be done to address climate change) linked to four clusters describing how climate change is framed by newspapers [25]. Additionally, Han et al. [26] documented how the Chinese government reframed climate change in the People’s Daily as an opportunity for better health and sustainable development. However, there was also a constant emphasis on economic growth in these stories. In an in-depth study of news articles in five European countries, Tavares et al. [27] identified 17 themes framing climate change. Regarding the theme of risk management, they note that almost half of the European news articles they reviewed did not mention specific actions and concluded that since media coverage emphasizes consequences that may occur in the distant future, the news media contributes to climate change apathy and disengagement. These studies demonstrate the potential importance of the framing of climate change discourse in its ability to influence climate change actions. Exploring the content of climate change-related discussions may provide an insight into why there is a gap between the expressed level of concern about climate change and actual behaviors to address climate change.

### 1.3. Social Media and Climate Change Discourse

Another approach to examining climate change discourse is through social media [28,29,30,31]. Social media posts on climate change have been analyzed for content and diffusion, and several studies have examined Twitter posts related to climate change. Tyangi et al. [32] reported that deniers of climate change on Twitter were more hostile toward people who believe in the human cause of climate change compared to those who do not. Several other studies have used machine learning via topic modeling with latent Dirichlet allocation to analyze climate-related Twitter posts. A study by Becken et al. [33] on 326,178 aviation and climate change tweets, using a machine learning approach, discovered that fairness and conflict frames (highlighting opposing views and behaviors) were most prominent, with high rates of retweets and denser networks, suggesting that messages containing opposing views were shared more widely.

Twitter may also promote sentiments hostile to addressing climate change. Tweets analyzed after Hurricane Sandy in 2012 showed several denial discourses, including rejecting climate science as a big government conspiracy, opposing renewable energy/energy taxation, and general fear of big governmental abuses of power [34]. While such studies provide valuable insight into frames, sentiments, and social network-based attitudes on climate change, they do not provide information on more common climate change conversations, which may differ from online discourse. Studies on Twitter are likely to suffer from bias and select individuals who are more engaged in climate change debates [35]. Moreover, Twitter exchanges may not involve close family and friends, who may be more influential than more socially distant individuals in influencing climate change attitudes and behaviors.

In the current study, we used a mixed-methods approach to examine whether individuals were having climate change-related conversations, as well as the factors associated with recent discussions about climate change. Among those who reported talking to others about climate change in the past month, we examined the content of these discussions and whether specific topics of conversation differed by demographic and political factors. Those who did not report the occurrence of climate-related conversations in the past month were asked to explain why such conversations did not occur.

One of the goals of the analyses was to gain potential insights into impediments to climate change conversations and the content of climate change conversations in order to promote social norms of engaging in actions to address climate change.

## 2. Methods

Study participants were drawn from the online longitudinal COVID-19 and Well-Being Study that began in March 2020. This study aimed to examine individual, social, and societal-level fluctuations associated with health and well-being amid the rapidly changing landscape of the pandemic. Respondents were assessed every 3–4 months. The initial study waves focused on COVID-19; however, as the pandemic continued, we assessed other global health issues and factors linked to health and well-being, including climate change.

Study participants were recruited online through Amazon’s Mechanical Turk (MTurk), a platform frequently used by health and social researchers. It allows for a diverse sample to be collected in a rapid and timely fashion [36]. Prior research suggests that MTurk provides better-quality data than other methods for recruiting convenience samples [37]. Study populations recruited through MTurk are not representative but have been documented to outperform other opinion samples on several dimensions [38]. Studies using MTurk have also demonstrated high levels of reliability [39]. The study protocols followed best practice guidelines for MTurk, including ensuring confidentiality, protecting study integrity, generating unique completion codes, integrating attention and validity checks throughout the survey, repeating study-specific enrollment qualification questions, and removing ineligible participants [37,40,41]. In addition, despite the COVID-19 pandemic, the demographic characteristics of MTurk appear stable [39]. The following criteria were used to determine eligibility: being age 18 or older, living in the United States, being able to speak and read English, having heard of coronavirus or COVID-19, and providing written informed consent. To augment validity, eligible participants were required to pass attention and validity checks embedded in the survey to mitigate inattentive and random responses [42,43]. These checks included survey questions with exceedingly low probabilities, such as having gone deep-sea fishing in Alaska and having appendages removed. We also repeated questions to ensure consistency. Finally, we examined the time participants took to complete the survey and verified survey completeness. Additional recruitment occurred at the fifth and sixth waves to increase sample diversity. Climate change was extensively assessed in wave six of the survey and served as data for the primary analysis of this study. Participants were compensated USD 4.25 for the sixth wave data (14–23 June 2021), which was equivalent to approximately USD 12 per hour. The survey was conducted in mid-June 2021, which was at the beginning of the wildfire season on the west coast and after the official start of the hurricane season on 1 June. But in 2021, major wildfires did not occur until later in the summer, and the first major hurricane was documented in August. There was a historic heath dome in the Pacific Northwest in 2021, with a four-day period with temperatures well over 100 degrees in cities such as Portland and Seattle. This extreme heat event occurred a few days after the survey (26 June).

The study protocols were approved by the University Institutional Review Board. In total, 1832 participants were screened, and 805 were eligible and passed the attention checks. A total of 15 respondents did not complete the survey and were excluded from the sample. The survey is included in the Supplementary Materials.

### 2.1. Qualitative Methods

The focus of the data analyzed in this study came from participant responses to the question, “In the last month, have you talked to a friend or family member about climate change?” Individuals who responded “yes” received the follow-up question, “Who did you talk to and what did you talk about?”, and those who selected “no” received the follow-up question, “Why didn’t you talk to anyone about climate change?” Thematic analysis was used to conduct the qualitative analysis [44]. Two research assistants independently familiarized themselves with the data and generated initial codes. The research team then met as a whole to review the coded segments and discuss any inconsistencies that arose between the two coders, revising the codebook as needed and recoding until the consistent application of all codes was achieved. Responses could be categorized into more than one code. Codes were then collated to identify the most salient themes within each coding category. In total, 786 of 805 (98%) participants responded to the open-ended questions. There were 33 participants (4%) who did not provide sufficient details to code (participants only reported whom they spoke to or “No reason”).

### 2.2. Quantitative and Mixed-Methods

To triangulate, generalize, and provide greater context and understanding, an embedded mixed-methods approach was used to perform quantitative and qualitative analyses on the dataset of the free-text responses and the factors associated with these responses. In the mixed-methods analysis, all themes from the qualitative analysis were included, except for the “other” theme due to the small size of the sample and salience. Information was also collected on the demographic factors of age, biological sex, education, race/ethnicity, political party affiliation, and income. Median splits were used for analyses of age, education, and income variables. Participants’ level of concern about climate change was assessed with the question, “How important is the issue of global warming to you personally?”. Response categories included “extremely important”, “very important”, “somewhat important”, “not too important”, and “not at all important”. Based on the distribution, the categories “not too important” and “not at all important” were collapsed.

After compiling descriptive statistics, chi-square tests were used in the quantitative analyses to examine demographic differences between those who talked about climate change in the prior month and those who did not. For the mixed-methods analyses, chi-square tests were used to compare those who discussed a specific theme in their conversations or reason for not talking about climate change to those who did not mention the theme or reason.

## 3. Results

A total of 805 participants responded to the question on whether they had recently discussed climate change and provided responses to the demographic questions. Most of the respondents were female (54.7%), age 40 or younger (58.4%), reported household incomes of USD 60,000 or less (55.5%), and had a bachelor’s degree or higher (58.6%). The largest proportion of respondents was white (65.6%), followed by Black (16.4%), Hispanic (9.2%), and Asian (5.7%). There were 504 (63%) respondents who reported that they did not have a climate conversation in the past month, while 301 (37%) reported that they did. Among the respondents who reported talking about climate change in the prior month, 63% reported talking about it a great amount and 37% reported a moderate amount.

As seen in Table 1, household income and education did not significantly differentiate those who had versus those who had not talked about climate change in the past month. Political party affiliation was associated with significant differences among those who did and did not discuss climate change. Among Democrats, 43.1% reported talking about climate change in the prior month, compared to 27.7% of Republicans and 37.6% of independents (*p* < 0.01). Age was marginally significantly associated with having discussions about climate change: 65.3% of respondents aged 18–40 did not discuss climate change, whereas 58.8% of respondents aged 41–90 did not discuss climate change in the prior month (*p* = 0.060). Although there was a strong association (*p* < 0.001) between ratings of the level of the personal importance of global warming and reports of talking about climate change in the prior month, among those who reported that global warming was extremely important, 35.2% had not talked about it in the prior month, and for those who reported that it was very important, the majority (56.4%) reported not discussing climate change in the prior month.

### 3.1. Qualitative Results

#### Climate Change Conversations

Among the 786 individuals who provided open-ended responses, there were five emergent themes identified among the topics of climate-related conversations and eight emergent themes among the reasons participants gave for not discussing climate change. Among those who did speak about climate change, themes included: (1) Weather (all codes related to changes in weather patterns and increased frequency of extreme weather events); (2) Impact (climate impact on future generations and impact on animals); (3) Actions (individual-level actions around climate change, policies/programs that could be enacted to address climate change, and outreach to elected officials); (4) Fault (describing who caused climate change); and (5) Other (non-specific descriptions of the climate change topic discussed). Table 2A,B include the final set of “Conversation” and “Non-Conversation” themes and codes.

1. Weather

The most common topics discussed among respondents who had climate conversations were those related to changes participants noticed in weather events, including wildfires, droughts, and other abrupt patterns. One participant shared:

*My family lives in Western Colorado and there was a forest fire in California. The amount that was burned was so much that you could smell the fire in Colorado. We talked about how global warming could be blamed for that. We talked about how the drought in Colorado is caused by global warming and getting worse every year.*

Another discussed noticing droughts, heat, and wildfires in their region of the country:

*It has come up in various conversations with friends, family and acquaintances, often regarding drought and heat in my part of the country (Southwest). Also, being a gardener, I’ve discussed the longer growing season and longer transitional seasons (spring and fall) with other growers. Additionally, we’ve talked about wildfires and the pall of smoke that we’ve been living in. I talk about EV’s constantly and renewable energy sources.*

Many participants also shared discussions about the increased frequency of extreme weather events, such as storms, hurricanes, and heatwaves. Several participants noted the unexpected winter storm in Texas as an example of this. Others highlighted the increasing frequency of hurricanes in Florida as an example of the impact of climate change:

*My mom about how hot the summers are and how cold the winters are getting year over year. And since I live in Florida, the number of hurricanes have increased every year since the year 2000.*

For many individuals in this group, observations of changing weather patterns and more extreme weather events led to climate change concern and prompted discussions among peers and network members.

2. Impact

Many participants also expressed an awareness of the adverse impacts of climate change on animals, ecosystems, and future generations. Several respondents reported discussing the effects of climate change on animals and the environment at large, highlighting the impact of climate change on animals, bees, melting ice caps, and rising sea levels. One stated:

*I talked with friends and family members about all sorts of things; ice melt, habitat loss of animals, migration of different species. Wildfires, drought, oceanic storms, many other things on the topic as well.*

Participants also expressed concern about the impact of climate change on animals in particular.

*I have talked to several people about the various changes we have been noticing due to climate change, the biggest concern would be the disappearance of the bees as we need them to survive, also how we never see butterflies anymore as well as certain birds that used to visit regularly.*

Others highlighted concerns about changes to ecosystems and environmental degradation, mentioning desertification, deforestation, melting glaciers, and algae blooms. One participant specifically highlighted changes in the ozone layer and awareness of melting polar ice caps:

*We were wondering about the ozone layer and if the hole that they said was in the ozone layer had gotten any bigger. All we hear now is about the polar ice caps melting.*

Due to the adverse impacts of climate change noted, several participants discussed their fears about the impact of climate change on future generations. One participant felt concerned enough to question whether she wanted to have children. She shared:

*My best friend and I talked about how I have decided that I may not want to have children anymore due to climate change. She has one kid, and does not think she will have more.*

For the individuals in this category, concerns about the impact of climate change prompted discussions about ecosystem loss, animal welfare, and future generations.

3. Actions

Some participants shared conversations about various actions that could be taken to mitigate the harmful impacts of climate change. Many participants discussed individual-level action steps they felt they could take to reduce the impact of global warming, including recycling, reducing one’s energy use, and spreading information about global warming to others within their networks. One participant reported several conversations with multiple network members about these action steps:

*I talked to my wife and best friend about ocean cleanup, I talked to my dad about alternative fuel systems and river cleanup, and I talked to my spouse and friends about habitat diversity. I also talked to my wife and sister about recycling.*

Another highlighted individual-level behaviors that were discussed with friends and family around “reducing our carbon footprint”, via “saving energy, water, recycling, use of public transportation, etc.”. (177) Only one participant mentioned engaging in outreach to elected officials regarding climate change action, stating:

*I send letters to those elected officials that represent me and my family and friends know where I stand on the issue. I also re-post significant information on Facebook.*

While most mentioned individual-level actions, several participants discussed an awareness of the need for broader, policy-level solutions to address climate change, highlighting that individual action alone would be insufficient to combat the issue. One shared:

*Just friends and family in general topical discussions. We all do our own things like recycle or turn off lights or don’t reproduce more humans. But ultimately we all know what we do is a drop in the ocean to what corporations and governments could do.*

Some participants noted policy-level solutions, particularly discussing the need for a community-level shift from fossil fuels to alternative fuel sources. One participant highlighted this, stating:

*I talked with my coworkers and we mostly addressed what type of energy plants we should enact as well as why we should enact them, especially nuclear. We also talked about the pros and cons of carbon tax and why it should be used on incentivizing people to switch to renewable energy sources and lowering taxes instead of punishing everyone.*

The individuals included in this category highlighted the importance of individual-level actions as a means to impact the adverse impacts of climate change.

4. Fault

Beyond discussions around noticing climate change and acknowledging its impacts, several participants reported discussing who was to blame for climate change. Conversations of this nature included beliefs that both corporations and humans were the causes of climate change through pollution, fossil fuel emissions, and land destruction. One participant shared:

*I talked to my kids about it and I let them know how our behavior as a nation is causing the current situation and will affect future generations*

Several individuals placed the blame of climate change on corporations, with one calling them “the biggest problem” in causing climate change. Another stated:

*I talked to my dad and my good friend. We talked about how weather is getting more severe in both directions. We talked about how the burden is often pushed onto us as individuals instead of corporations and industrial farms that do 99% of the damage.*

Within this group, participants acknowledged the role of both individuals and larger corporations in damaging the environment through climate change.

5. Other

The largest group in the “other” theme did not provide specific details of their conversation. Under the code of general conversations were statements such as, “we just talked about the issue generally” and “I talked to my sister and we just had a general conversation about climate change”.

The 18 respondents (6%) coded under the “other” theme as “climate change is exaggerated or not real”, “climate change used as a political tool”, or “misunderstanding of climate change, e.g., not an issue” all expressed a sentiment that climate change was not human-caused, real, or a threat.

### 3.2. Why Climate Change Was Not Discussed

For those participants who reported that they had not discussed climate change in the past month, a follow-up question prompted them to report why such conversations had not been had. Responses fell into several themes, including (1) Denial (how the effects of climate change are exaggerated, climate change is a hoax/not a real issue, or misunderstandings/misinformation about climate change); (2) Not a priority (comprising all codes related to other priorities that took precedence, or being too busy to be concerned about climate change); (3) Will not make a difference (including reasons discussions are ineffective); (4) Difficult to discuss (including reasons why it is difficult to bring up the climate change topic); (5) Not an issue cared about (including all codes related to individuals not caring about climate change or stating it was not something they worried about); (6) Not enough time (too busy); (7) Did not come up; and (8) Other.

1. Denial

A sizable number of respondents reported not talking about climate change because of disbelief in the issue. Responses in this category ranged from some who felt climate change effects were exaggerated, to others reporting it to be a full-on hoax. One stated:

*Because I do not think its real. its just another way for the government to try and control us. Making the American people fight each other over nothing. Life is too short to be that serious about something that is blown out of the water so crazily.*

Among those who felt climate change was not real, several reported the belief that alarm about climate change has been generated as a political ploy:

*It isn’t an issue at all. Climate change used to be called global warming (now debunked), then global freezing, then it became climate change because it can’t prove the previous results. It is political for money subsidies for failing development of green technologies.*

Further, some participants reported believing that a false hysteria had been created around climate change in order to benefit big industries, including the technology sector, to create “green” products. Others felt changes in climate were no different than any previous climate shifts in the Earth’s history, stating:

*I don’t care and view it more as climate shifting within earth’s natural cycle of changing patterns in heat and cold that happen every few hundred years*

Overall, mistrust of government and a faulty understanding of climate change informed the attitudes of those who do not acknowledge climate change as an issue.

2. Not a priority

Many respondents who did not discuss climate change felt that the issue was not a priority in their lives. Many cited other issues that took precedence, including family, work, and the COVID-19 pandemic. One shared:

*I think regular life… pandemics, work, children, even sports and puppies take precedence.*

(166)

Others described climate change as taking a backseat to issues that they deal with daily. Climate change effects have been slowly occurring over decades, and the impacts are often invisible on a day-to-day basis. Several participants highlighted this, either describing that they did not personally see the impacts of climate change or would not within their lifetimes. One stated:

*I don’t care. I don’t have a wife or children so there will be nobody in the future that are a part of my progeny that will be affected by climate change. Plus, I hate talking to and dealing with people in general. Let them suffer the consequences of their own actions, I won’t do anything to help them.*

Such apathy towards climate change presents a major challenge to engagement in climate change-related discussions.

3. Will not make a difference

Another group of respondents largely believed in climate change but did not think discussing the topic would have any impact. Many in the group felt that institutions, such as corporations and governments, bear the burden of climate change, and therefore discussing it with peers would be a waste of time. One participant asserted:

*The major problem with Climate Change is the big corps, and unless forced to stop, they will continue to mess everything up. The problem isn’t you, or your everyday person. Others still contribute to it, yes, but a majority of it comes from the corps.*

This group reported little faith in the power of collective action and expressed that individual action is futile to combat climate change, whether this action is focused on reducing its impact or convincing others to care about climate change. One participant stated:

*Why would I? People either understand the science or they do not. Most people are not undecided due to lack of information. They believe what they want to. Nothing I say is going to change that.*

Individuals in this group express the inability of individuals to meaningfully intervene in the global effects of climate change.

4. Difficult to discuss

Many participants reported finding it difficult to discuss climate change among members of their networks. Some found the topic to be too contentious to want to bring it up in conversation. In addition, some individuals believed that their peers were not interested in discussing climate change or felt that climate change was not a natural part of their regular conversations, so conversations about climate change would be poorly received. One participant shared:

*It’s not an issue my friends/family really discuss very often. I also find this issue to be very polarizing so it’s not much different than discussing politics or religion. Lastly, I wouldn’t consider myself to be very well informed on the matter.*

Many felt that bringing up climate change would lead to contentious debates and wished to avoid discussing controversial topics with their networks, stating:

*They do not like talking about controversial topics. They do not want to be lectured. They told me that once when we were talking about immigration.*

Again, due to “contentiousness”, climate change was not discussed. Responses also revealed that although an individual may want to engage in climate change discussions, those around them could be opposed.

5. Not an issue they cared about

Many respondents reported that they did not discuss climate change because it is either a subject that does not interest them or one that they do not view as a critical issue in their life. These responses reflect a lack of urgency to address climate change. Some responses included:

*Because it’s a boring subject and nobody cares, and, It’s not a major concern to me; and thus, not worth discussing.*

Climate change was unimportant to this group of respondents.

6. Not enough time

Several participants reported that they did not discuss climate change because they are too preoccupied with the activities of their daily life. One shared:

*I’m really busy and it just doesn’t normally come up in conversation.*

Others did not have time to broach the subject of climate change within their social groups. Another stated:

*I’ve been too busy with work and taking care of my elderly mom. I don’t have time for socializing or much less anything else the last few months.*

Similar to the group of respondents who had other priorities in their life, this group typically does not have time to address climate change in their daily lives.

7. Did not come up

A large number of respondents reported not discussing climate change because it was not a topic of conversation amongst their peers. One shared:

*It doesn’t come up in my circles.*

Another participant stated:

*I didn’t talk to anyone about it because it was never a topic that came up in my conversations and it isn’t an issue that my friends or family talk about often.*

Responses from these participants demonstrate the importance of social networks in generating climate change conversations.

8. Other

A few respondents reported that they did not discuss climate change for a range of reasons less common among all the respondents. A few reported generally not speaking with many people or avoiding discussing social issues with others. One shared:

*Because I don’t often talk to people in general*

Another participant stated:

*I don’t discuss my personal opinions about these topics*

The largest portion of this group reported no specific reason for not discussing climate change.

### 3.3. Mixed-Methods Analyses

Most responses (90.1%) were classified into only one theme among those who provided reasons for which they did not talk about climate change, with 9.5% coded as two themes and 0.6% with three themes. Among those who did report climate change conversations, 90.7% of responses were classified into one theme and 9.3% into two themes.

The first set of inferential statistical analyses examined differences between those respondents who had talked about climate change in the prior month and those who had not.

We conducted 66 chi-square tests (11 themes and 6 demographic factors) to assess the relationship between themes and participants’ demographic characteristics. Completed data were available for 485 respondents who had not talked about climate change and 298, out of 301, who had talked about it in the prior month. Out of the 66 chi-square tests, only 10 were statistically significant, indicating that demographics were only associated with select themes of climate change communication topics and reasons for not discussing climate change.

Themes of the climate change conversation topics only significantly differed by political affiliation. Political party affiliation was associated with the themes of denial (Republicans, 16.3%; Democrats, 1.0%; Independents, 8.7%%, χ^2^ = 26.42, *p* < 0.01) and actions to address climate change (Republicans, 7.1%; Democrats, 22.5%; Independents 13.0%, χ^2^ = 8.03, *p* < 0.05).

Themes representing reasons for not talking about climate change in the prior month were significantly different by select demographic characteristics. Respondents who reported having a higher education status were more likely to report that climate change was not a priority (16.7% vs. 24.6%, χ2 = 4.24, *p* < 0.05). Income was only associated with the themes that climate change was difficult to discuss and climate change denial. Respondents who reported a higher income were less likely to report that climate change was difficult to discuss (5.5% vs. 13.2%, χ^2^ = 8.17, *p* < 0.05) and were more likely to report climate change denial (11.9% vs. 6.9%, χ^2^ = 5.15, *p* < 0.05). Race was associated with the following themes: climate change was difficult to discuss, climate change denial, and climate change not being an issue they cared about. Whites were more likely to indicate denial as a reason for not discussing climate change compared to non-whites (10.9% vs. 5.2%, χ^2^ = 6.17, *p* < 0.05), whereas non-whites were more likely to report that they did not talk about climate change because it was difficult to discuss compared to whites (13.9% vs. 7.5%, χ^2^ = 5.16, *p* < 0.05). Whites were also more likely than non-whites to report that climate change was not an issue they cared about (28.1% vs. 16.4%, χ^2^ = 8.22, *p* < 0.05). Gender was not associated with any topic of conversation or reasons for not discussing climate change. Age was associated with the theme of denying climate change as a reason for not discussing the issue, with a larger proportion of older respondents denying climate change (12.0% vs. 5.6%, χ^2^ = 8.35, *p* < 0.05). Among the reasons individuals did not talk about climate change, there was only one statistically significant difference: political party was associated with reporting that the topic of climate change did not arise (Republicans, 25.5%; Democrats, 38.5%; Independents 28.6%, χ^2^ = 7.18, *p* < 0.05).

## 4. Discussion

In this study, we employed a mixed-methods approach to explore whether individuals had participated in recent climate change-related conversations, exploring specific conversation topics and the reasons climate change was not discussed. Most respondents reported that they had not discussed climate change in the prior month. Even among individuals who reported that the issue of global warming was “extremely important” to them personally, over one-third had not had a conversation about climate change in the prior month. The most frequently reported reasons cited for which climate was not discussed included that climate change was not a priority, that participants did not care about it, and that the topic did not arise in conversation. The latter category of not coming up in conversations was reported by almost one-third (31%), suggesting that climate change may not be a normative discussion topic for many people. A smaller proportion (9%) of those who did not discuss climate change indicated that they were climate change deniers. Denial was more prevalent among older respondents, which has been found in other studies [45,46,47]. 

We did not find that demographic factors were strongly associated with topics of climate change conversations or the reasons why respondents did not talk about climate change. One explanation for this lack of association is that other factors may have a greater impact on the frequency and content of climate change conversations. Moreover, some of the conversations about climate change documented in this paper may occur as a means of social discourse to exchange pleasantries, such as conversations about the weather, or to affirm one’s identity as someone concerned about climate change, and hence are not linked to demographic factors.

Among those respondents who had climate conversations, the most common topic discussed was the weather. This finding aligns with prior research that suggests that assessments of weather patterns and events are associated with perceptions of climate change [48]. Studies in the literature describing this relationship tend to find that people who have experienced the impact of climate change, such as extreme weather events, also tend to believe that climate change is occurring. Of 32 papers that address the association between self-reported weather experiences and climate change opinions, 27 (84%) found evidence for such a relationship [48]. For example, a large study of surveys from 119 countries found that perceptions of rising temperatures at the local level predicted climate change risk perceptions [49]. However, there is much more mixed evidence for the relationship between objective measures and climate change opinions [50].

Given the finding of the high frequency of weather-related climate change discussions, it may be useful for environmental organizations to increase lobbying efforts following extreme weather events before they either fade in salience or are pushed out of the news by more immediate issues. However, one problem with making climate change more salient through discussions about recent extreme weather events is that people quickly begin to view new temperature patterns as normal and, hence, less salient [51]. It is also likely that there will be less media attention paid to dangerous weather events that are not record-breaking, despite having tremendous impacts on human and environmental well-being.

Although climate change discussions may be linked to weather events, there is no evidence from the current study that these conversations are coupled with conversations about behaviors to mitigate the impact of climate change. This is consistent with prior research suggesting that a large proportion of the US population is concerned about climate change, yet few are engaged in activities to address climate change mitigation [3]. To facilitate actions to address climate change, discussions about climate change should include conversations about what citizens can do to address climate change, which, in turn, may lead to action. An important empirical question is how conversations about weather events can be actively steered away from simply acknowledging the changes toward a discussion about what individuals can actually do about these events.

Very few individuals discussed potential actions that could be taken to address climate change. There may be several reasons why respondents did not discuss individual or collective action to address climate change. These may include a lack of knowledge about how to engage in climate change action or a lack of confidence in the potential impact of actions to help address climate change. Future research should further explore the factors that impede individuals from engaging in climate-related actions. It should be noted that respondents were far more likely to report discussing individual-level actions such as recycling compared to political actions to address climate change. This perspective may be partly due to the US being a highly individualistic culture, which has led to a societal framing that environmental behaviors should be individual acts rather than determined by social factors and legal policies.

One pathway through which climate change conversations may lead to action is through changing social norms. Discussions about the climate may influence opinions and behaviors to become more salient, leading to descriptive norms that others are concerned about climate change and/or engaged in activities to address it. Evidence suggests that descriptive social norms are associated with climate change actions of voting, donating, volunteering, contacting government officials, and protesting [52]. Social norms may also impact why individuals refrain from climate change conversations. Others not raising the topic of climate change may lead to perceptions that the issue is not important. One hypothesis is that climate-related conversations are not happening due to the concept of the “spiral of silence”, which suggests that some people may not talk about climate change because they are concerned about what other people might think about their opinions. There are mixed findings on the role of the spiral of silence regarding opinions about climate change, but future research should explore the concept in the context of common climate change conversations [53,54]. The current study did not find evidence of a “spiral of silence”; only ten respondents (2%) indicated that they did not talk about climate change because it was too contentious or political. Even if a lack of climate change conversations is not due to a spiral of silence, climate change conversations may not be normative for many people. Therefore, it may be useful to provide people with suggestions on how to raise the topic of climate change so that it becomes normative.

Although there is evidence that climate change activists have conversations with family and friends to encourage collective actions, there is little information about the most effective approaches [55]. To motivate those individuals who are highly concerned about climate change and talk about it yet are not engaged in actions to address it, environmental organizations could highlight this gap and emphasize that many actions, such as calling and writing local and national policymakers, require minimal time and resources. One impediment to engaging in climate change actions in many countries is that the issue has become politically polarized. In the US, due to the hyperpartisan nature of the political debate regarding climate change, it may be valuable to not only lobby federal, state, or local representatives but also have conversations about climate change with social network members who have different political party affiliations and encourage them to engage in climate action. It has been documented within the US that Democrats perceive Republicans to be more anti-climate change action than they actually are [56,57]. This dynamic may be due to a lack of conversations about climate change among members of different political parties, which may result in further inaction at a political level. Fostering more frequent climate change conversations may help to address this misperception.

The limitations of this study should be noted. This was not a random sample; thus, our findings may not generalize to the broader US population with different socio-demographic characteristics or populations outside of the US. We also do not know the accuracy of the recollection of climate change conversation topics or how specific residential locations, or residential mobility, may have affected the responses. Future studies should ask when the conversation occurred to examine how time since the conversation may influence memories of the topic. The timing of the survey may have also influenced conversation topics. The COVID-19 pandemic may have made the topic of climate change less salient and less of a priority, and the timing of the survey in relation to extreme weather events may have also influenced conversation frequencies and content. However, even if climate change conversations become more frequent after extreme weather events, evidence from this study does not suggest that an increase in conversations will lead to actions to address climate change given that the majority of conversations do not include discussion of collective action to mitigate climate change. These findings suggest that policymakers and environmental organizations should work to foster public discourse that facilitates collective action to address climate change. Future studies may benefit from examining how the content of activists’ conversations differs from that of individuals who are not involved in climate change activism and with whom that leads to activism.

## 5. Conclusions

The findings from this study suggest that climate change is not a frequent topic of conversation among many US residents, and when it is discussed, it is often in regard to weather events. Few everyday climate change conversations include discussions of engagement in either individual or collective action to address climate change. Clear and consistent messages about what citizens can do to direct conversations to lead to action, which promotes policymakers addressing climate change, are needed. Environmental organizations should consider training their members in communication skills to bridge the gap between concern about climate change and engagement in collective action to address it.

## Figures and Tables

**Table 1:** Participant characteristics by engaging in climate conversation in past month (n = 805).

	Talked Last Month N (%)	Didn’t Talk Last Month N (%)	Total Sample N (%)	Chi-Square	*p*-Value
Sex				1.92	0.165
Male	127 (34.8%)	238 (65.2%)	365 (45.3%)		
Female	174 (39.5%)	266 (60.5%)	440 (54.7%)		
Race/Ethnicity				1.80	0.773
White	197 (37.3%)	331 (62.7%)	528 (65.6%)		
Black	53 (40.2%)	79 (59.8%)	132 (16.4%)		
Hispanic	29 (39.2%)	45 (60.8%)	74 (9.2%)		
Asian	14 (30.4%)	32 (69.6%)	46 (5.7%)		
Other	8 (32.0%)	17 (68.0%)	25 (3.1%)		
Education				1.24	0.266
Less than bachelor’s degree	117 (35.1%)	216 (64.9%)	333 (41.4%)		
Bachelor’s degree or higher	184 (39.0%)	288 (61.0%)	472 (58.6%)		
Income				1.77	0.675
USD 60,000 or less	170 (38.0%)	277 (62.0%)	447 (55.5%)		
More than USD 60,000	131 (36.6%)	227 (63.4%)	358 (44.5%)		
Age				3.54	0.060
18–40	163 (34.7%)	307 (65.3%)	470 (58.4%)		
41–90	138 (41.2%)	197 (58.8%)	335 (41.6%)		
Political party				13.175	0.004
Republican	57 (27.7%)	149 (72.3%)	206 (25.6%)		
Democrat	152 (43.1%)	201 (56.9%)	353 (43.9%)		
Independent	77 (37.6%)	128 (62.4%)	205 (25.5%)		
Other	15 (36.6%)	26 (63.4%)	41 (5.1%)		
How important is the issue of global warming to you personally?				138.70	<0.001
Extremely important	140 (64.8%)	76 (35.2%)	216 (27.8%)		
Very important	99 (43.6%)	128 (56.4%)	227 (28.2%)		
Somewhat important	41 (20.4%)	160 (79.6%)	201 (25.0%)		
Not too or not at all important	21 (13.0%)	140 (87.0%)	161 (20.0%)		

**Table 2:** (**A**) Frequency themes and codes among participants who discussed climate change (298); (**B**) frequency of themes and codes among participants who did not discuss climate change.

**(A)**				
**Theme**	**Codes**	**N**	**%**	**Total**
Weather	Changes in weather patterns and events over time: length of seasons, drought, wildfires, etc.	72	24.2	298
	Increased frequency of extreme weather: hurricanes, heat waves, etc.	34	11.4	298
Impact	Impact on future generations: not having more kids, frequency of disease	12	4.0	298
	Effects on animals and environment: impact on animals, bees, extinction, ozone, ice caps melting, rising sea levels	21	7.0	298
	Important issue/worried	8	2.7	298
Actions	What individuals can do (not political actions): recycling, lifestyle, sharing information on social media, etc.	39	13.1	298
	Outreach to elected officials via letters	1	0.03	298
	Policies/programs that should be enacted: alternative fuel sources, public transit	13	4.0	298
Fault	Corporations cause climate change	6	2	298
	Human caused climate change: pollution/fossil fuel emissions, destroying land	15	5	298
Other *	General conversation	50	16.8	298
	Non-specified topic: participant only reported whom they spoke to	17	5.7	298
	Climate change is exaggerated/not real	6	2.0	298
	Survival	2	0.7	298
	Cannot do anything to solve climate change	3	1	298
	Climate change used as a political tool	4	1.3	298
	Frustration with climate change deniers	3	1	298
	Efforts to educate others	6	2	298
	Misunderstanding of climate change, e.g., not an issue	9	3	298
(**B**)				
**Theme**	**Codes**	**N**	**%**	**Total**
Denial	Effects of climate change are exaggerated	7	1.4	485
	Not a real issue/hoax	31	6.4	485
	Misunderstanding of climate change, e.g., not an issue	10	2.1	485
Not a priority	COVID more pressing issue	22	4.5	485
	Climate change not a priority: personal life, family, other social issues more important	70	14.4	485
Will not make a difference	Discussions are ineffective/will not change anything	23	4.7	485
	We know where we stand on the issue, so don’t discuss	14	2.9	485
Difficult to discuss	Not informed enough to confidently discuss climate change or educate others	16	3.3	485
	Contentious topic/too political	10	2.1	485
	Too hard to bring up (because topic is uncommonly discussed)	4	0.8	485
	Other people don’t want to talk about it	20	4.1	485
Not an issue cared about	Not an issue they care/worry about/find important	98	20.2	485
	Don’t care about it	13	2.7	485
	Not personally impacted	7	1.4	485
Not enough time	Too busy	15	3.1	485
Did not come up	Topic did not come up in conversation	149	30.7	485
Other *	Don’t talk to people often	2	0.4	485
	No reason	16	3.3	485
	Other	9	1.9	485

* This theme was excluded from quantitative analyses due to low frequencies and low saliency.

## Data Availability

The datasets generated and analyzed during the current study are not publicly available due to ongoing study, but they are available from the corresponding author upon reasonable request.

## Supplementary Materials

The following supporting information can be downloaded at: https://www.mdpi.com/article/10.3390/ijerph21030279/s1, Survey.
