# Supplementary material for: ‘They Talk about the Weather, but No One Does Anything about It’: A Mixed-Methods Study of Everyday Climate Change Conversations
Source: Int J Environ Res Public Health. 2024 Feb 28;21(3):279. doi: 10.3390/ijerph21030279 (PMC10970140; doi:10.3390/ijerph21030279)
Supplement: Supplementary file 1 [file ijerph-21-00279-s001.zip › ijerph-2767744-supplementary.pdf]

End of Block: Consent

Start of Block: mTURK ID

Q2 Please enter your mTurk ID:

____

End of Block: mTURK ID

Start of Block: Section I: Demographics

Q3 **How old are you?**

____

Page Break

Q4 **Have you moved states since coronavirus (mid-March 2020)?**

○ Yes (1)

○ No (2)

Page Break

Display This Question:

If Have you moved states since coronavirus (mid-March 2020)? = Yes

Q5

What state do you live in now?

▼ Alabama (1) ... Washington D.C. (51)

Page Break

Display This Question:

If Have you moved states since coronavirus (mid-March 2020)? = Yes

Q6 **What size community do you live in now?**

- 1. Large urban area (>250,000 residents) (1)
- 2. Medium urban area (100,000-250,000) (2)
- 3. Town (2,500 to under 100,000) (3)
- 4. Rural (under 2,500) (4)

Page Break

Q7 **What sex were you assigned at birth, on your original birth certificate?**

- ○ Male (1)
- ○ Female (2)

Page Break

Q8 **What is your race/ethnicity?**

- ○ 1. White (1)
- ○ 2. Non-Hispanic Black (2)
- ○ 3. Hispanic (3)
- ○ 4. Asian (4)
- ○ 5. Mixed (5)
- ○ 6. Other (6)

Page Break

Q9 **What is the highest level of education you've completed?**

- 1. Grade 11 or less (1)
- 2. Grade 12 or GED (2)
- 3. Some college, Associate's Degree, or Technical Degree (3)
- 4. Bachelor's Degree (4)
- 5. Graduate degree (MD, PhD, Masters) (5)

Page Break

Q10 **Are you currently required to report to work outside of your home?**

- ○ Yes (1)
- ○ No (2)

Page Break

Q11 **What is your relationship status?**

- 1. Married (1)
- 2. In a committed relationship (2)
- 3. Single (3)
- 4. Divorced; separated (4)
- 5. Widowed (5)

Page Break

Q12 **Has your living situation changed since coronavirus (mid-March, 2020)?**

- Yes (1)
- No (2)

Page Break

Display This Question:

If Has your living situation changed since coronavirus (mid-March, 2020)? = Yes

Q13 **What best describes your current living situation?**

- 1. Live in a house that I own (1)
- 2. Live in a house or an apartment I rent (2)
- 3. Rent a room in someone else's house or apartment (3)
- 4. Stay with someone else for free (4)
- 5. Live on the street or in a shelter (5)
- 6. Stay at more than two different places a week (6)
- 7. Live in a recovery or transitional house (7)
- 8. Other place (8)

Page Break

Q14 **How many people currently live in your household?** This means children or adults (including yourself) who sleeps in the house at least two nights or more every week.

○ # of people (1) ____

Page Break

*

Q15 **How many children do you have under the age of 18 living in your household?** This means children who sleep in the house at least two nights or more every week.

○ # of children (1) ____

Page Break

Display This Question:

If If How many children do you have under the age of 18 living in your household? This means children w... # of children Is Not Equal to 0

Q16 **Do any of your children qualify for free/reduced meals at school?**

- Yes (1)
- No (2)

Page Break

Q216 **This next set of questions asks about global warming and climate change.**

Q217 **How important is the issue of global warming to you personally?**

- Extremely important (1)
- Very important (2)
- Somewhat important (3)
- Not too important (4)
- Not at all important (5)

Page Break

Q218 **How worried are you about global warming?**

- ○ Extremely worried (1)
- ○ Very worried (2)
- ○ Somewhat worried (3)
- ○ Not worried at all (4)

Page Break

Q219 **How much do you think global warming will harm you personally?**

- ○ A great deal (1)
- ○ A moderate amount (2)
- ○ Only a little (3)
- ○ Not at all (4)

Page Break

Q220 **How much do you think global warming will harm future generations of people?**

- ○ A great deal (1)
- ○ A moderate amount (2)
- ○ Only a little (3)
- ○ Not at all (4)

Page Break

Q221 **Please rate the reasons you haven't been involved in climate change advocacy.**

	Yes (1)	No (2)
Too busy (1)	o	o
Don't know how to get involved (2)	o	o
Other people are better at it than me (3)	o	o
If I do get involved, organizations will ask me for money (4)	o	o
Haven't been asked (5)	o	o
Activities like letter writing aren't appealing (6)	o	o
I haven't been trained (7)	o	o
Not encouraged to become involved (8)	o	o
Not something I think about a lot (9)	o	o
What I could do will not have an impact (10)	o	o
Other people might react negatively to my involvement (11)	o	o
I don't think it's a problem (12)	o	o
Not a priority for me (13)	o	o

Page Break

Q222 **In the last year, have you donated money to an organization to address climate change?**

- ○ Yes (1)
- ○ No (2)

Page Break

Q223 **In the last year, have you contacted an elected official about climate change?**

- Yes (1)
- No (2)

Page Break

Q224 **In the last month, have you talked to a friend or family member about climate change?**

- Yes (1)
- No (2)

Page Break

Display This Question:

If In the last month, have you talked to a friend or family member about climate change? = No

Q225 **Why didn't you talk to anyone about climate change?**

____

____

____

____

____

Page Break

Display This Question:

If In the last month, have you talked to a friend or family member about climate change? = Yes

Q226 **Who did you talk to and what did you talk about?**

____

____

____

____

____

Page Break

Q227 **If you saw this image on a plastic container, what would it mean to you?**

- ○ It definitely can be recycled (1)
- ○ It probably can be recycled (2)
- ○ It most likely cannot be recycled (3)
- ○ I cannot tell if it can be recycled (4)
- ○ Other (please specify): (5) ____
- ○ Don't know (6)

Page Break

Q228 **When I see a number that refers to recycling on a plastic container, I usually don't know what the number means.**

- ○ Strongly agree (1)
- ○ Agree (2)
- ○ Neither agree nor disagree (3)
- ○ Disagree (4)
- ○ Strongly disagree (5)

Page Break

Q229 **A number less than 6 on a plastic container means that it can be recycled.**

- ○ Strongly agree (1)
- ○ Agree (2)
- ○ Neither agree nor disagree (3)
- ○ Disagree (4)
- ○ Strongly disagree (5)

Page Break

Q230 **Do you consider yourself a Republican, Democrat, Libertarian, or other?**

- Republican (1)
- Democrat (2)
- Independent (3)
- Libertarian (4)
- Other (5)

Page Break

Q231 **Where would you place yourself on a scale running from "very liberal" to "very conservative?"**

- Very liberal (1)
- Liberal (2)
- Slightly liberal (3)
- Moderate (4)
- Slightly conservative (5)
- Conservative (6)
- Very conservative (7)
- Not applicable (8)

Page Break

Display This Question:

If Do you consider yourself a Republican, Democrat, Libertarian, or other? = Republican

Or Do you consider yourself a Republican, Democrat, Libertarian, or other? = Democrat

Q232 **Being a Democrat/Republican is a very important part of who I am.**

- Strongly agree (1)
- Agree (2)
- Neither agree nor disagree (3)
- Disagree (4)
- Strongly disagree (5)

Page Break

Q233 **What percent of your friends are Republicans?**

- 0-10% (1)
- 10-20% (2)
- 20-30% (3)
- 30-40% (4)
- 40-50% (5)
- 50-60% (6)
- 60-70% (7)
- 70-80% (8)
- 80-90% (9)
- 90-100% (10)

Page Break

Q234 **What percent of your friends are Democrats?**

- 0-10% (1)
- 10-20% (2)
- 20-30% (3)
- 30-40% (4)
- 40-50% (5)
- 50-60% (6)
- 60-70% (7)
- 70-80% (8)
- 80-90% (9)
- 90-100% (10)

Page Break

Q235 **I strongly dislike the Republican party.**

- Strongly agree (1)
- Agree (2)
- Neither agree nor disagree (3)
- Disagree (4)
- Strongly disagree (5)

Page Break

Q236 **I strongly dislike the Democratic party.**

- Strongly agree (1)
- Agree (2)
- Neither agree nor disagree (3)
- Disagree (4)
- Strongly disagree (5)

Page Break

Q237 **Do you think of yourself as gay, lesbian, straight, bisexual, something else, or you don't know the answer?**

- ◯ Gay (1)
- ◯ Lesbian (2)
- ◯ Straight (3)
- ◯ Bisexual (4)
- ◯ Something else (5)
- ◯ You don't know the answer (6)

Page Break

Q238 **How often do you go to religious services?**

- Every week (1)
- Several times a month (2)
- A few times a year (3)
- Never (4)

Page Break

Q239 **Do you consider yourself evangelical or born again?**

- ◯ Yes (1)
- ◯ No (2)

Page Break

Q240 **What was the total income for your family last year?**

- ○ Less than $15K (1)
- ○ $15K to $35K (2)
- ○ $35K to $60K (3)
- ○ $60K to $90K (4)
- ○ $90K and over (5)
